# Topographical relocation of adolescent sleep spindles reveals a new maturational pattern in the human brain

**DOI:** 10.1038/s41598-022-11098-8

**Published:** 2022-04-29

**Authors:** Ferenc Gombos, Róbert Bódizs, Adrián Pótári, Gábor Bocskai, Andrea Berencsi, Hanna Szakács, Ilona Kovács

**Affiliations:** 1 Laboratory for Psychological Research, Pázmány Péter Catholic University, 1 Mikszáth Kálmán Sq., Budapest, 1088 Hungary; 2 Adolescent Development Research Group, Hungarian Academy of Sciences - Pázmány Péter Catholic University, Budapest, 1088 Hungary; 3 Institute of Behavioural Sciences, Semmelweis University, Budapest, 1089 Hungary; 4 National Institute of Clinical Neurosciences, Budapest, 1145 Hungary; 5 Doctoral School of Mental Health Sciences, Semmelweis University, Üllői st. 26, Budapest, 1085 Hungary; 6 Institute for the Methodology of Special Needs Education and Rehabilitation, Bárczi Gusztáv Faculty of Special Needs Education, Eötvös Loránd University, Budapest, 1097 Hungary; 7 Institute of Cognitive Neuroscience and Psychology, Research Centre for Natural Sciences, Budapest, 1117 Hungary

**Keywords:** Sleep, Neuroscience, Non-REM sleep, Electroencephalography - EEG

## Abstract

Current theories of human neural development emphasize the posterior-to-anterior pattern of brain maturation. However, this scenario leaves out significant brain areas not directly involved with sensory input and behavioral control. Suggesting the relevance of cortical activity unrelated to sensory stimulation, such as sleep, we investigated adolescent transformations in the topography of sleep spindles. Sleep spindles are known to be involved in neural plasticity and in adults have a bimodal topography: slow spindles are frontally dominant, while fast spindles have a parietal/precuneal origin. The late functional segregation of the precuneus from the frontoparietal network during adolescence suggests that spindle topography might approach the adult state relatively late in development, and it may not be a result of the posterior-to-anterior maturational pattern. We analyzed the topographical distribution of spindle parameters in HD-EEG polysomnographic sleep recordings of adolescents and found that slow spindle duration maxima traveled from central to anterior brain regions, while fast spindle density, amplitude and frequency peaks traveled from central to more posterior brain regions. These results provide evidence for the gradual posteriorization of the anatomical localization of fast sleep spindles during adolescence and indicate the existence of an anterior-to-posterior pattern of human brain maturation.

## Introduction

Details on the protracted maturational course of the human brain have been accumulating due to advanced brain imaging technologies^1–3^, and the characteristic back-to-front pattern has become a dominant presumption with respect to large-scale structural maturation^4^. According to the latest accounts of adolescent functional connectivity, the association areas or the so-called default mode network (DMN) of the brain are remodeled during development at an even later age than those areas that are more related to immediate sensory input and behavioral control^5,6^. While the latest maturing hot-spots in the network subserving “internal” cognition are believed to be fundamental to human evolution^7–9^, their exact connectivity and contribution to adolescent development is not known. We suggest that the investigation of spontaneous cortical activity in the absence of sensory stimulation, such as sleep, might help to move this field forward, and a more complete account of the developing large-scale functional networks will emerge.

The dynamics of large-scale brain networks are tightly linked with neural oscillatory activities. Sleep spindle oscillations are groups of 11–16 Hz waves emerging in nonrapid eye movement (NREM) sleep resulting from the rhythmic hyperpolarization rebound sequences of thalamocortical neurons^10,11^, the inhibition of which is caused by the NREM-dependent activation of GABAergic neurons in the reticular thalamic nucleus. Sleep spindles emerging from spontaneous neural activity during sleep reflect the underlying large-scale cortical functional networks^12^. A bimodal frequency and topography of sleep spindles has been described in adults. Slow (~ 12 Hz) sleep spindles are frontally dominant, whereas fast (~ 14 Hz) sleep spindles are of parietal/precuneal origin in adults^13,14^.

The precuneus is a major hub of the DMN, part of the complex neurocognitive network involved in the maintenance of conscious awareness, self-reflection, visuospatial integration and episodic memory^9,15^. Similar to the frontal cortex, precuneus expansion is a neurological specialization of Homo sapiens and is assumed to be associated with recent cognitive specializations^16^. The precuneus has been characterized by increasing functional segregation from the frontoparietal network between 8 and 26 years of age^17^. In addition to the late maturation of frontal lobe functions characterized by the well-known back-to-front maturational pattern of the brain^2–4,18^, the precuneus is also a subject of significant developmental changes in adolescence.

Based on the relatively late functional segregation of the precuneus from the frontoparietal network, as well as on the bimodal prefrontal versus precuneal sources of slow and fast sleep spindles in the adult brain, we postulate that sleep spindles undergo a topographical transformation characterized by increasing anatomical segregation during adolescent development. We assume that slow and fast sleep spindles are gradually relocated from the more central toward the prefrontal and parietal regions, respectively. Age-related topographical transfer is assumed to reflect the increasing differentiation and segregation of the frontal and precuneal subsystems in the DMN and the associated late development of phylogenetically new neuroanatomical structures in humans.

Here, we focus on the late maturation of slow frontal and fast parietal sleep spindle features in adolescence by using high-density electroencephalography (HD-EEG) recordings in three subgroups of different ages (12, 16 and 20 years). We hypothesize that the two types of sleep spindles index distinct neurodevelopmental processes during the course of adolescent brain maturation and functional specialization. We believe that focusing on the bimodal antero-posterior source and frequency distribution of sleep spindle oscillations^13,14^, contrasting the unimodal, frontal maxima of slow waves^19,20^ provides us with a better understanding of adolescent remodeling of the human brain.

## Results

We recorded 128 channel full-night sleep HD-EEG data and detected individual-specific slow and fast spindles by the Individual Adjustment Method (IAM, see Methods/Procedure and ref.^21^). We analyzed the derivation-specific topographical distribution of the spindle parameters (density, duration, maximum amplitude and peak frequency) for NREM sleep. The distribution of the spindle parameters along the midline derivations (from Fpz to Iz) was calculated, and the maximum position of the distribution for every spindle parameter was determined. We scrutinized anterior–posterior shifts of the maximum locations as a function of age.

**Figure 1:**
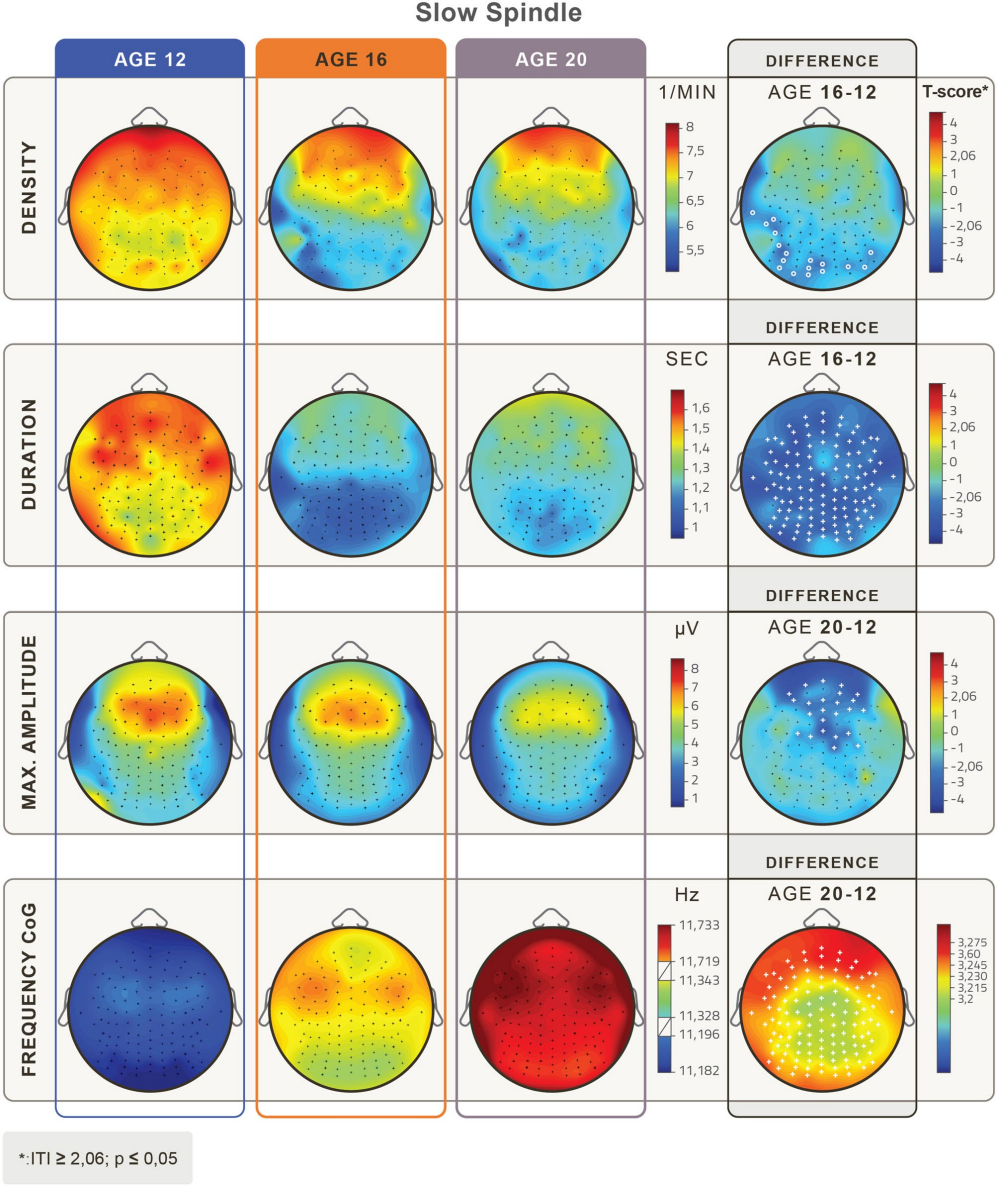
Topographical distribution of mean slow sleep spindle parameters in the age groups of 12, 16 and 20 years of age. Rows depict age-related changes in slow sleep spindle density (1/min), duration (s), maximum amplitude (µV), and frequency based on the center of gravity (CoG) (Hz). Relevant age-group differences are depicted in the 4th column. White crosses depict Rüger area significant differences and white circles depict significant but not Rüger area significant differences. Age-related decreases in density are significant with an occipital and left temporal focus. There was a significant overall decrease in duration and a frontal-frontopolar decrease in maximum amplitude. Frequency increases at all derivations with a frontal focus from the younger to the older age groups. (Level of significance: p < .05, T-score values are matched with the colorbar, T ≥ │2.06│ *p* < 0.05, at T ≥ │2.787│ *p* < 0.01 and at T ≥ │3.725│ *p* < 0.001).

**Figure 2:**
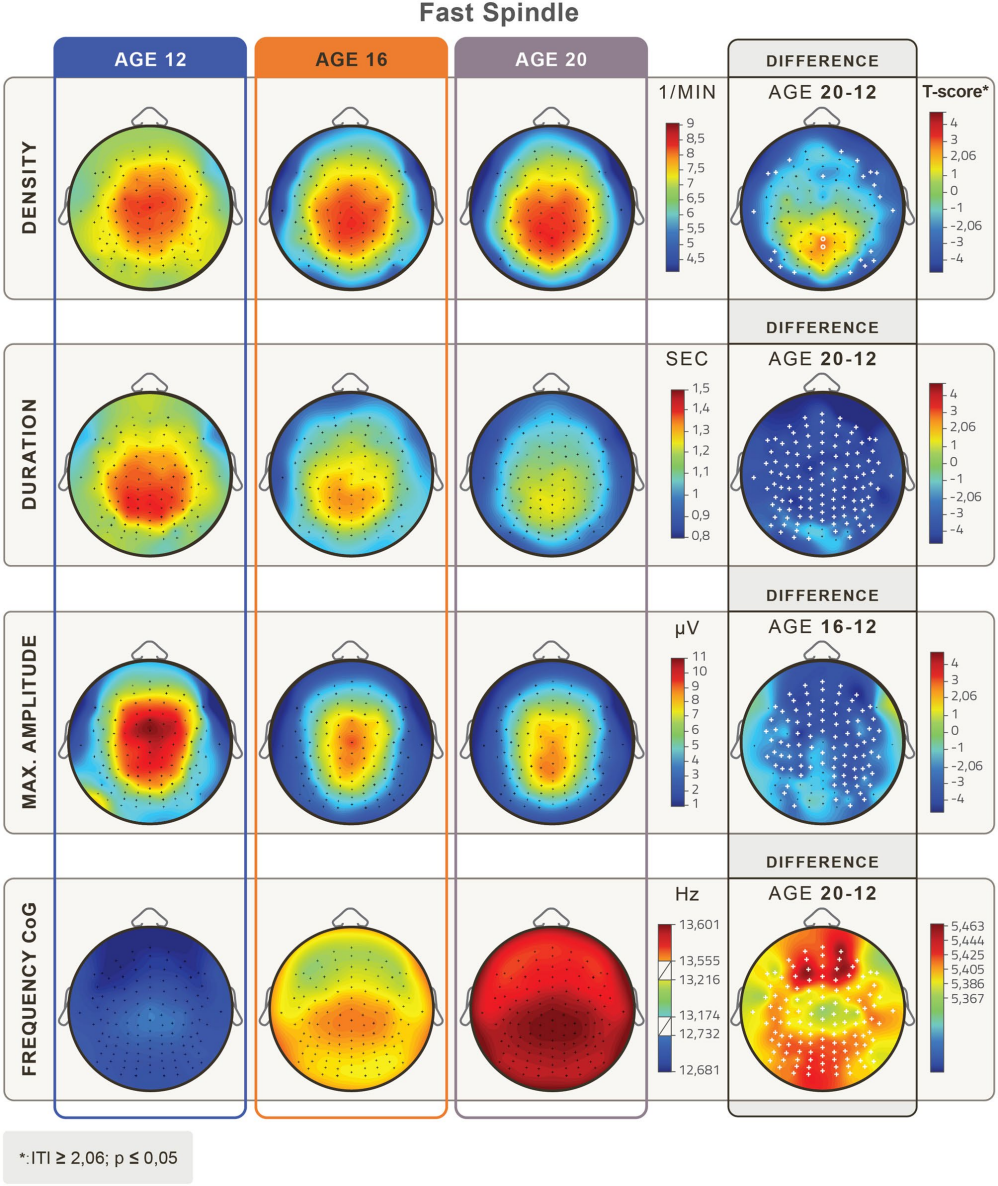
Topographical distribution of mean fast sleep spindle parameters in the age groups of 12, 16 and 20 years of age. Rows depict age-related changes in fast sleep spindle density (1/min), duration (s), maximum amplitude (µV), and frequency based on the center of gravity (CoG) (Hz). Relevant age-group differences are depicted in the 4th column. White crosses depict Rüger area significant differences, and white circles depict significant but not Rüger area significant differences. Fast spindle density increased significantly, but not Rüger significantly centro-parietally between 12 and 20 years of age, and Rüger significantly decreased at the perimeter of the scalp. There was a significant overall decrease in duration and a frontal decrease in maximum amplitude. Frequency significantly increased at all derivations with frontocentral maxima from the younger to the older age group. (Level of significance: *p* < .05, T-score values are matched with the colorbar, T ≥ │2.06│ *p* < 0.05, at T ≥ │2.787│ *p* < 0.01 and at T ≥ │3.725│ *p* < 0.001).

**Figure 3:**
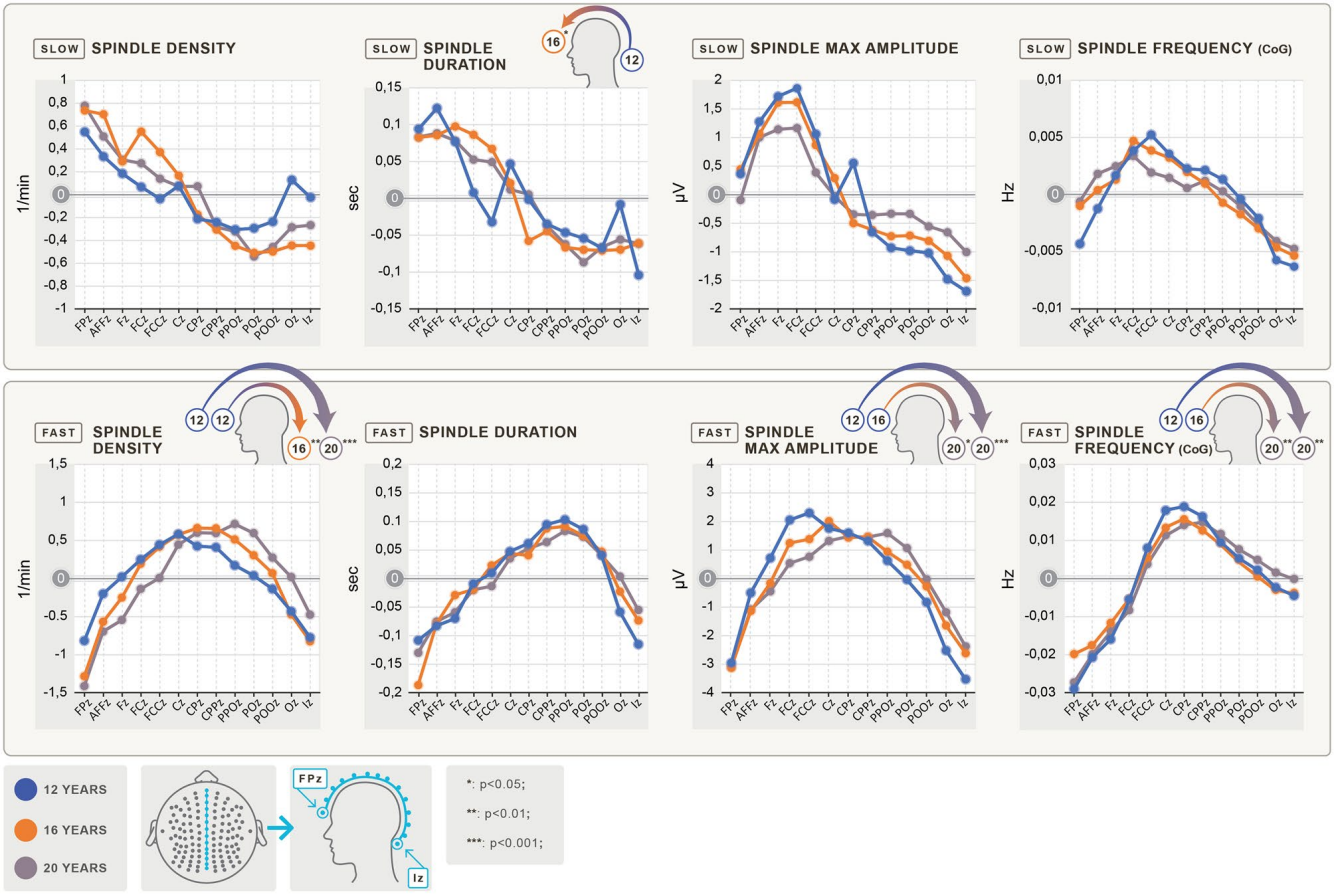
Age-related antero-posterior shifts in sleep spindle parameters. The spindle parameter values (y-axis) for the midline electrodes (Fpz to Iz on the x-axis, from left to right from frontal to occipital) are plotted by age groups. As the differences are significant but small (differences due to spatial position are much smaller than differences between age groups), the y-values display the difference from the mean value of the midline electrodes of the specific age group. The upper row depicts slow spindle parameters, while the lower row depicts fast spindle parameters. The different columns show different spindle parameters, such as density (1/min), duration (sec), maximum amplitude (µV) and frequency (Hz), based on the center of gravity (CoG). The small head figures above the graphs indicate significant differences and the direction of the difference in the positions of the maximum values between the specific age groups. Blue indicates 12-, orange indicates 16- and gray indicates 20-year-old subjects. Slow spindle parameters tended to shift toward the anterior region, whereas fast sleep spindle parameters tended to shift toward the posterior regions with age. The shift of the maximum values of slow spindle duration toward frontal regions was statistically significant. In terms of fast spindle density, maximum amplitude and frequency, the shift of the maximum values from central to centroparietal regions was statistically significant.

Here, we first describe the topographical distribution and age-related topographical changes of slow and fast spindle parameters (Figs. 1 and 2). Second, we present evidence for age-related antero-posterior shifts in sleep spindle parameters (Fig. 3).

### Topographical distribution and age-related topographical changes of slow and fast spindle parameters

The age-dependent topographical distributions of slow and fast spindle parameters are shown in Figs. 1 and 2, respectively.

In terms of duration, the decrease in slow spindles from age 12 to 16 was Rüger area significant for all recording locations (T = (− 2.05–− 3.29), *p* = (0.002–0.04)). Maximum amplitude values of slow spindles decreased with frontal and frontopolar foci between all age groups from 12 to 20 years (T = (− 2.03–− 2.99), (*p* < 0.001–0.048)). Frequency increased Rüger area significantly at all derivations with a frontal focus throughout the entire adolescent period (T = (3.2–3.28), *p* = (0.002–0.003)), see Fig. 1.

Fast spindle density decreased Rüger area significantly at the perimeter of the scalp (T = (− 2.07–− 3.06), p = (0.04 – 0.004)). Fast spindles revealed a significant decrease in duration for all recording derivations over the entire adolescent period from age 12 to 20 (T = (− 2.06–− 4.54), (*p* < 0.001–0.047)). Fast spindle maximum amplitude values decreased significantly in all areas with a frontal and central focus (T = (− 2.05–− 4.54), (*p* < 0.001–0.048)). Fast spindle frequency values increased with age. The differences are Rüger area significant for the entire cortex in all age groups (T = (5.37–5.46), *p* < 0.001), see Fig. 2.

### Age-related shifts in sleep spindle parameters

Topographical shifts of slow and fast spindle parameters can be visually assessed in the topographical distribution maps of Figs. 1 and 2, respectively. While slow spindle parameters tended to shift toward anterior electrode positions, fast sleep spindle measures seemed to shift toward posterior electrode positions with age. To more precisely quantify these observable tendencies, we calculated the distribution of spindle parameters along the midline derivations (from Fpz to Iz) and determined the maximum position of the distribution for every spindle parameter.

Maximum values of slow spindle duration shifted toward frontal electrode positions. The difference in the positions between the age groups of 12 and 16 years of age was statistically significant (F(1,38) = 4.3; *p* = 0.045; ANOVA). In terms of fast spindle density, the difference in positions of maximum values was significant in the age groups of 12 to 16 (F(1,38) = 11.27; *p* = 0.002; ANOVA) and 12 to 20 (F(1,38) = 23.84; *p* < 0.001; ANOVA), revealing a central to centroparietal shift with age. Maximum values of the positions of fast spindle maximum amplitudes shifted significantly from central to centroparietal electrode positions in the age groups of 16 to 20 (F(1,38) = 4.5; p = 0.04; ANOVA) and 12 to 20 (F(1,38) = 13.2; *p* < 0.001; ANOVA) but not between ages 12 to 16. Maximum values of the positions of fast spindle frequency also shifted significantly from central to centroparietal electrode positions in the age groups of 16 to 20 (F(1,38) = 11.76; *p* = 0.001; ANOVA) and 12 to 20 (F(1,38) = 9.77; *p* = 0.003; ANOVA) (Fig. 3). SI Fig. 1. shows age-related mean positions of the maxima of sleep spindle parameters along the antero-posterior axis in the midline EEG recording locations. Positions of the maxima of fast spindle density, maximum amplitude and frequency age-dependently relocate from central (12 years) to parietal (20 years) regions, whereas slow spindle duration express their maxima in more anterior regions in the 16 and 20 years old subjects as compared to the 12 years group (shifts forward from Fz to AFFz).

## Discussion

Here, we report evidence for distinct shifts in the predominant location of slow and fast sleep spindle oscillations during adolescent development. We found evidence for slow sleep spindle duration maxima traveling from central to anterior brain regions and fast spindle density, amplitude and frequency peaks traveling from central to more posterior brain regions. These findings coincide with reports of an age-related increase in the functional segregation of the precuneus (a major source of fast sleep spindle oscillations in humans^13,14^) from the frontoparietal region from childhood to adulthood^17^.

In the late maturing frontal and parietal cortical areas, the short-range local connections are dominated by long-range functional networks during development^22^. In the course of adolescence, the anterior–posterior connectivity of the frontal and parietal lobes increases in the DMN^23,24^. Although functional connectivity may be reduced in the DMN in deep sleep stages^25,26^, it is preserved to such an extent that oscillatory activity-related functional connectivity between anterior and posterior areas corresponds to DMN characteristics during sleep spindling^27^.

Previous HD-EEG reports, based on sleep slow waves, have shown the anteriorization of sleep state-dependent neural oscillations during human ontogenesis (including the period of adolescence)^18^; however, age-related posteriorization of any frequency band has not been shown before. Thus, the late maturation of the frontal lobes is clearly supported by findings of former sleep neurophysiological studies, as well as our present report on slow sleep spindle oscillation; however, the phylogenetically new precuneal region has not yet been targeted by such studies. Although it could have been assumed that the late maturing precuneus is characterized by age-related posteriorization of sleep-state-specific oscillations, indexing the timing of developmental plasticity, such findings have been obscured by the insufficient frequency resolution of former reports.

Studies investigating the development of sleep spindles in children or adolescents do not cover the age-dependent topographical maturation of sleep spindles due to the low number of EEG electrodes^28–30^. Here, we use an individually adjusted frequency analysis of sleep spindle oscillations, with high frequency resolution and 128 channel HD-EEG recording, which is sufficiently sensitive to capture this process. Recent findings indicate the need of increased sensitivity and decreased specificity of sleep spindle detection procedures, as sleep spindles comprise a subset of a broader class of electroencephalogram events^31^. IAM was shown to be characterized by increased sensitivity and decreased specificity compared with other sleep spindle detection methods^32^. With these more sensitive methods, we have been able to show that individual-specific fast sleep spindle features unequivocally travel to more posterior areas during adolescence. Considering the precuneal origin of fast sleep spindles in adult human subjects^13,14^, it seems that individualized sleep spindle analysis is a forceful tool to detect the dominant anatomical directions of brain maturation in humans.

Prevailing concepts of complex neural development and brain maturation emphasize the posterior-to-anterior or back-to-front pattern^2–4^ as an unequivocal ontogenetic feature, finding its roots in the late maturing frontal lobes. The back-to-front pattern has received substantial support from HD-EEG studies examining the age-related changes in the topography of sleep slow waves^18^. However, the above scenario of unidirectional developmental and anatomical route disregards the major hub of the DMN, which is a human-specific and late maturing neural structure of the brain, undergoing significant differentiation during adolescence. In addition to slow waves, sleep spindles are the major hallmarks of NREM sleep, known to be involved in neural plasticity with major sources in the precuneus, at least in healthy adult human subjects. Here, we provide evidence for a backward move in the anatomical localization of fast-type sleep spindles; that is, the predominantly central maxima in density and amplitude at the age of 12 years gradually become replaced with parietal electrode locations by the age of 20 years. Similar changes, albeit at a smaller anatomical distance, take place in terms of fast sleep spindle frequency. These findings complete the back-to-front pattern of brain maturation and cohere with reports of increased differentiation of the precuneus during adolescence. To the best of our knowledge, this is the first report of an age-related front-to-back transfer of the maxima of a neural plasticity-related sleep EEG index.

Direct cortical records of sleep spindles in children of prepubertal/early pubertal ages revealed prevailing perirolandic sources^33^ potentially indicating the initial maturational stage before the accomplishment of the topographical relocation described in our present study. Furthermore, the findings of Zerouali et al. (2014)^34^ indicate that precuneal, posterior sources are potential initiators of overlapping fast and slow sleep spindle complexes, providing a leading role of this area in off-line, adult-type distributed neural network processing reflected by such overlapping wave sequences. Although we did not specifically analyze spindle co-occurrences in our study, such analyses could reveal the dynamic properties of topographical distinction and perhaps cortical maturation and specialization during adolescent development. We have to note however, that according to earlier studies the subpopulation of overlapping slow and fast sleep spindles represent only 5% of all spindles^35^. We aim to elucidate these details in our future studies.

Apparently, adolescence is a timeframe of significant changes in the EEG spectra, including shifts in sleep spindle characteristics^28–30,36^. Sleep spindles have outstanding relevance from a clinical point of view since adolescence-related psychiatric disorders such as schizophrenia^37^, attention deficit hyperactivity disorder (ADHD)^38^ and depression^39^ have all been associated with anomalies in sleep spindles. It has also been suggested that sleep spindle abnormalities are an endophenotype of schizophrenia^37,40^. Moreover, these disorders can also be characterized by cognitive impairments^41–43^. It has been demonstrated that sleep spindles and cognitive function are strongly associated^44,45^, and as such, sleep spindle abnormalities might provide a key to interpreting cognitive malfunction^10^. Further investigating the mechanisms and developmental trajectory of sleep spindles in adolescence could lead to a better understanding of the aforementioned disorders.

Although genetic factors play a pivotal role in shaping the individual-specific parameters of slow and fast sleep spindle frequency activities^46^ and sleep spindles themselves^47^, we were able to detect meaningful age group effects, suggesting that the individualization of the parameters (IAM) combined with HD-EEG provides a powerful strategy in capturing developmental processes in the sleep neurophysiology domain. The reported differences in the contributions of genetic factors to slow and fast sleep spindle parameters are controversial**;** however, the almost perfect night-to-night stability of these measures is unequivocal^47,48^. That is, cross sectional studies are plausible approaches in unravelling the maturational routes in sleep spindles. Complementing the inferences of a cross-sectional study with inferences from a longitudinal design could map out a more precise developmental trajectory and further reduce the bias from individual differences.

A limitation of the current study is that analysis of EEG data can only provide indirect evidence for the precuneal origin of fast spindles. Source localization analysis has been beyond the scope of the current study, but applying such techniques would provide more convincing evidence for the link of the topographical shift of fast spindle frequency peaks and the ontogenetic development of the precuneus.

In conclusion, we focused on the late maturation of individually adjusted slow-frontal and fast-parietal sleep spindle features in adolescence by using HD-EEG recordings. Our hypothesis assuming that the two sleep spindle types might index distinct neurodevelopmental processes during the course of adolescent brain maturation and functional specialization has been confirmed by the data. We are convinced that focusing on the bimodal antero-posterior source and frequency distribution of sleep spindle oscillations has taken us closer to a better understanding of adolescent remodeling of the human brain.

## Methods

### Participants

We recruited 60 adolescent and young adult participants (30 females and 30 males, mean age 16.55 ± SD 3.70) via social media in three age groups, with an equal number of females and males in each age group: 12-year-olds (n = 20, mean age = 12.45 ± SD 0.57 years), 16-year-olds (n = 20, mean age = 15.91 ± SD 0.48 years), and young adults referred to as 20-year-olds (n = 20, mean age = 21.29 ± SD 0.51 years). Subjects with an existing neurological condition or sleep disorder were not included in the study. All subjects received a voucher in the value of HUF 20,000 (cca. USD 70.00) for their participation. The study was approved by the Ethical Committee of the Pázmány Péter Catholic University for Psychological Experiments. Adult participants and parents or legal guardians of the underage participants signed informed consent for the participation in the study according to the Declaration of Helsinki.

### Procedure

We performed 128 channel HD-EEG polysomnographic (PSG) sleep recordings of two consecutive nights in the Sleep Laboratory of Pázmány Péter Catholic University Budapest. We requested participants to maintain a regular sleep–wake schedule for 5 nights before recordings, but we did not monitor compliance. We asked participants not to consume any drugs other than contraceptives and coffee and to refrain from napping on the afternoons of the days of the experiment. Participants with any history of sleep problems or mental disorder or any neurological or medical condition were excluded. We verified such criteria by personal interviews and questionnaires completed by the parents or young adult participants themselves. We applied electrodes for electroencephalography (EEG), electromyography (EMG) and electrooculography (EOG). After checking the electrodes for skin contact and impedance levels, all participants went to sleep in the laboratory between 10.00 p.m. and 11.30 p.m. according to their preference. They slept until they awoke spontaneously.

We used a built-in EEG channel-Quick Cap (Compumedics, Australia) with 128 electrodes in three different head sizes to record EEG signals. We roughly evenly spaced the passive Ag/AgCl electrodes down to line T9-Iz-T10. The monopolar channels were referenced to a frontocentrally placed ground electrode. We applied bipolar EOG and EMG channels to measure eye movements and muscle tone, respectively, and placed the EOG channels below and to the left of the left eye (1 cm below the left outer canthus) and above and to the right of the right eye (1 cm above the right outer canthus). We determined the locations of the EMG electrodes in accordance with the recommendations of the American Academy of Sleep Medicine (AASM)^49^. We recorded the data using a BQ KIT SD LTM 128 EXPRESS (2 X 64 channels in master and slave mode, Micromed, Mogliano Veneto, Italy) recording device and subsequently visualized, saved and exported them using System Plus Evolution software (Micromed, Mogliano Veneto, Italy). We recorded all HD-EEG, EOG and EMG data with an effective sampling rate of synchronous 4096 Hz/channel with 22 bit equivalent resolution and prefiltered and amplified them with 40 dB/decade high- and low-pass input filters of 0.15–250 Hz. We applied a digital low pass filter at 231.7 Hz and downsampled the data to 512 Hz/channel by firmware.

From the recorded nighttime EEG recordings, the current analysis deals with the second night data as that was less impacted by adaptation effects. We manually scored sleep states of 20 s epochs of whole night NREM EEG recordings by visual inspection according to standardized criteria^50^ and revised hypnograms. Then, we removed artifacts on four-second long epochs and scanned the whole-night recordings four times to overcome the limitations of the capacity of the visual inspector (only 32 channels of the 128 were visually inspected in a run), and finally, we excluded any epoch marked as artifact in any run from further analysis.

We detected individual-specific slow and fast spindles by using the Individual Adjustment Method (IAM)^21^. The theoretical background of the IAM relies on the thesis that sleep spindles are the wave segments which make up the individual-specific spectral peaks in the NREM sleep EEG spectra. After removing sleep spindles detected by the IAM, the spectra of the non-spindle parts of NREM sleep EEG is of aperiodic, fractal-type nature, with no spectral peaks in the 9–16 Hz range. The other theoretical point of IAM finds its roots in the reports indicating spatially non-continuous antero-posterior changes in sleep spindle frequencies. As regarding the details of the IAM, the latter consists of the following main steps:

1. Calculating the amplitude spectra of artifact-free NREM sleep EEG by FFT for all available EEG locations.
2. Calculating the second derivatives of the 9–16 Hz spectra at all EEG recording locations and averaging them over locations (this results in one averaged second derivative).
3. Selecting the individual slow and fast sleep spindle boundaries by looking for zero crossings of the averaged second derivative surrounding negative peaks (positive peaks are turned to negative in the second derivatives).
4. Defining individual- and EEG location-specific amplitude criteria of slow and fast sleep spindles by linearly interpolating the amplitude spectra between the specific frequencies determined above.
5. Band-pass filtering the artifact-free MREM sleep EEG time series at individual-specific slow and fast sleep spindles separately, determining the envelopes of the slow- and fast sleep spindle filtered signals and applying the above determined amplitude criteria for slow and fast sleep spindles at each EEG location.

The individual fast and slow spindle frequency ranges and the midpoints of the frequency ranges for the 60 subjects included in the analyses are illustrated in SI Fig. 2. A scheme of spindle detection is provided in Supplementary Fig. 3. In fact, these segments contribute to the individual- and derivation-specific lower- and higher-frequency spectral peaks between 9 and 16 Hz. Based on the IAM approach, we determined individual and derivation-specific densities (spindles × min − 1), durations (s), and amplitudes (µV) of slow, frontally dominant and fast, centro-parietally dominant sleep spindles.

We determined the peak frequency using the center of gravity (CoG) of the individualized spectral peak frequencies in the spindle range (11–16 Hz). We obtained values of CoG by using the method described in the study conducted by Dustman^51^ In general the CoG is the average location or the balance point of all the weight of an object. In our case it is the average frequency location of the sleep spindle activity. It is calculated based on the spectrum of the EEG. CoG frequency for a given sleep spindle type (slow or fast) of a given channel of a given subject is calculated by:

$$CoG \, freq = \mathop \sum \limits_{{n = {\text{MinFreq}}}}^{{{\text{MaxFreq}}}} \left( {n*Amp_{n} } \right)/\mathop \sum \limits_{n = \min freq}^{\max freq} \left( {Amp_{n} } \right)$$

where MinFreq equals to the lower threshold (rounded up to the nearest 1/16 Hz) and MaxFreq equals to the upper threshold (rounded down to nearest 1/16 Hz) of the individual frequency range of the given spindle type. The step size in the sum is 0.0625 Hz (1/16 Hz) due to the resolution of the spectrum. Amp_n_ equals to the amplitude of the spectrum at frequency n of the given channel of the given subject and it represents the weights in the equation. We excluded outliers from the original data set by using Tukey's fences method^52^. This method is based on the quartiles of the data, the outliers are determined by the interquartile ranges. Let Q_1_ be the lower quartile and Q_3_ the upper quartile. The values outside the range [Q_1_-k(Q_3_-Q_1_), Q_3_ + k(Q_3_-Q_1_)] are considered outliers. The value of k is traditionally chosen to be 1.5 (outlier) or 3 (far out). We used the more stringent value of 1.5 in our analysis.

We analyzed the derivation-specific topographical distribution of spindle parameters and their age-dependent differences. We used a t-test to compare the activity of the corresponding electrodes between each group. In order to provide a control of multiple testing and the consequent inflation of type I error, the procedure of descriptive data analysis^53^ adapted to quantified neurophysiology with mapping^54^ was applied. This procedure tests the global null hypothesis (“all individual null hypotheses in the respective region are true”) at level α = 0.05, against the alternative that at least one of the null hypotheses is wrong. According to Abt^53^ and Duffy et al.^54^ local, uncorrected and spatially contiguous significances at the level of α = 0.05 (descriptive significances) define the Rüger’s areas^55^. Areas with fewer than three adjacent derivations are not included in the Rüger significant regions. If N is the number of electrodes in the Rüger’s area, the investigator is required to choose a minimal number of unspecified null hypotheses (M), less than N, to be nominally rejected at a new, more conservative α level. Typically the value M/N is 1/2 or 1/3. The corresponding new α levels for these values are α/2 = 0.025 and α/3 = 0.017, respectively. We will use M/N values of 1/2 and 1/3 with corresponding new α levels of 0.025 and 0.017, respectively. If any M values (half of the t-tests if M/N = 1/2 and third of the t-tests if M/N = 1/3) within the Rüger’s area individually reach the new α level of significance (0.025 and 0.017, respectively) the overall null hypothesis is rejected for the Rüger’s area at the 0.05 level. This means that for at least one EEG derivation in the Rüger’s area the relationship is significant, allowing the investigator to make global confirmatory statement with controlled uncertainty.

Finally, we calculated the distribution of the spindle parameters along the midline derivations (from Fpz to Iz) and determined the maximum position of the distribution for every spindle parameter. We examined the anterior–posterior shifts of the maximum locations as a function of age. Statistical comparisons were conducted by one-way ANOVA models (one for each spindle parameter) with the anteroposterior location of the spindle parameter as the dependent variable and age group as the categorical independent variable.

For the processing of sleep data (sleep state classification, artifact rejection and spindle detection with IAM), we used the FerciosEEGPlus 1.3 program (http://www.gomboshaz.hu/fercioseegplus.htm). We used Microsoft Excel for the calculation of CoG values and Rüger area corrections. We conducted all statistical tests using TIBCO STATISTICA 13.5.0.17 software (TIBCO Software Inc., 2018).

## Supplementary Information

Supplementary Information.

## Data Availability

The datasets analyzed during the current study are available from the corresponding author on request.
