# Supplementary material for: Topographical relocation of adolescent sleep spindles reveals a new maturational pattern in the human brain
Source: Sci Rep. 2022 Apr 29;12:7023. doi: 10.1038/s41598-022-11098-8 (PMC9054798; doi:10.1038/s41598-022-11098-8)
Supplement: Supplementary file 1 — Supplementary Information. [file 41598_2022_11098_MOESM1_ESM.pdf]

Supplementary information

for

Topographical relocation of adolescent sleep spindles reveals a new maturational pattern in the human brain

Ferenc Gombos [a, b, †], Róbert Bódizs [c, d, †], Adrián Pótári [b], Gábor Bocskai [a,e], Andrea Berencsi [b, f], Hanna Szakács [a,e] and Ilona Kovács [a, b, g, *]

[a] Laboratory for Psychological Research, Pázmány Péter Catholic University, Budapest, 1088 Hungary
[b] Adolescent Development Research Group, Hungarian Academy of Sciences - Pázmány Péter Catholic University, Budapest, 1088 Hungary
[c] Institute of Behavioural Sciences, Semmelweis University, Budapest, 1089 Hungary
[d] National Institute of Clinical Neurosciences, Budapest, 1145 Hungary
[e] Doctoral School of Mental Health Sciences, Semmelweis University, Üllői st. 26, Budapest, 1085 Hungary
[f] Institute for the Methodology of Special Needs Education and Rehabilitation, Bárczi Gusztáv Faculty of Special Needs Education, Eötvös Loránd University, Budapest, 1097 Hungary
[g] Institute of Cognitive Neuroscience and Psychology, Research Centre for Natural Sciences, Budapest, 1117 Hungary

[†]F.G. and R.B. contributed equally to this study and should be considered joint first Authors

*Corresponding author: Ilona Kovács, Laboratory for Psychological Research, Pázmány Péter Catholic University, 1 Mikszáth Kálmán sq., Budapest, 1088 Hungary
Email: dr.ilona.kovacs@gmail.com

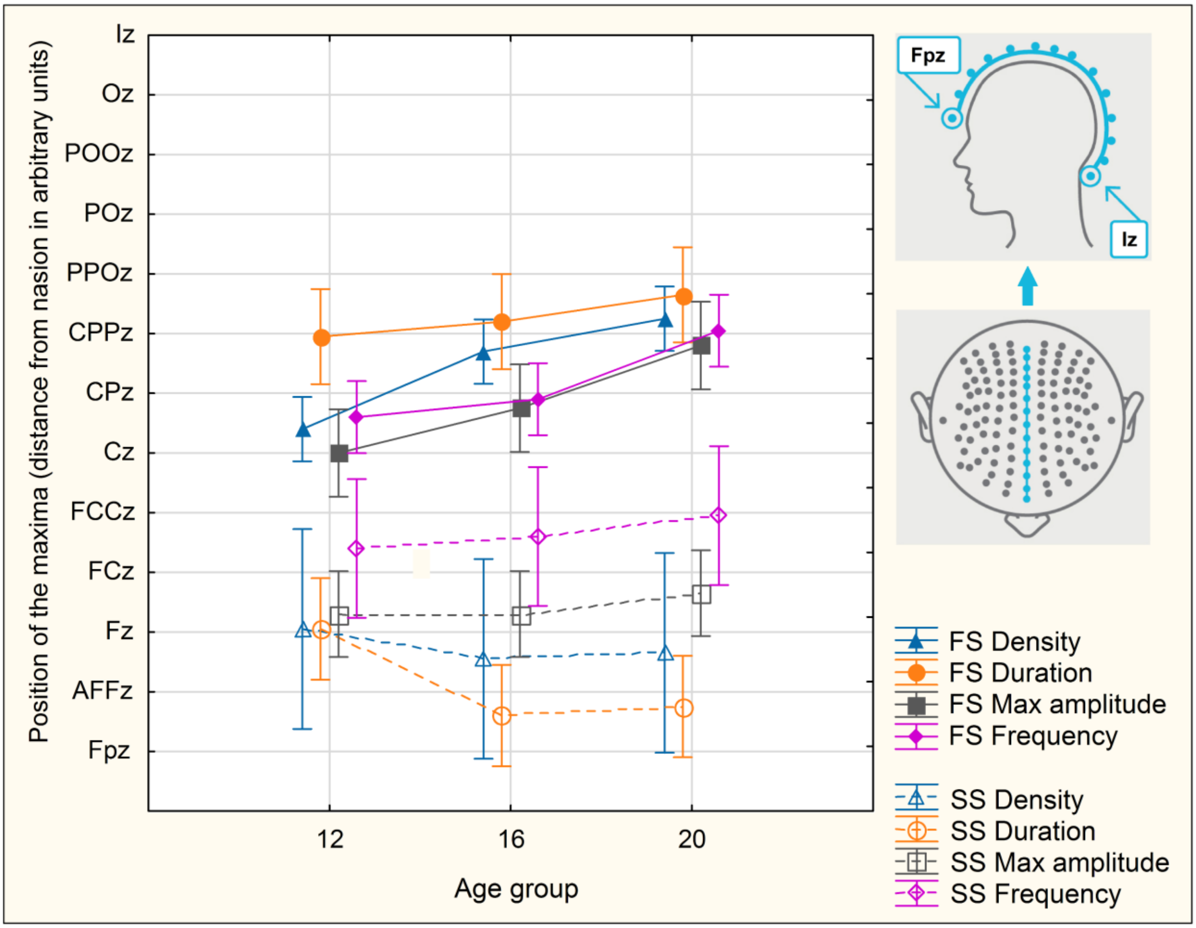

Supplementary Figure 1. Mean position of the maxima of sleep spindle parameters along the midline in different age groups. Topographical locations of the individual-specific maxima of different sleep spindle parameters along the midline (sagittal) channel-row were numerically transformed in order to quantify anteriority/posteriority of the respective measures (Fpz = 1, AFFz = 2, ... , Iz = 13, higher values indicate more posterior locations). Group means (empty and filled markers) and 95% confidence intervals (vertical bars) of these measures were calculated for both slow spindle (SS) and fast spindle (FS) parameters, whereas vertical scale labels back-transformed from numbers to the original EEG location symbols. Positions of the maxima of fast spindle (FS) density, maximal amplitude and frequency are characterized by significant age-dependent shifts in the posterior direction, while position of the maxima of slow spindle (SS) durations relocates in more anterior regions during adolescent development.

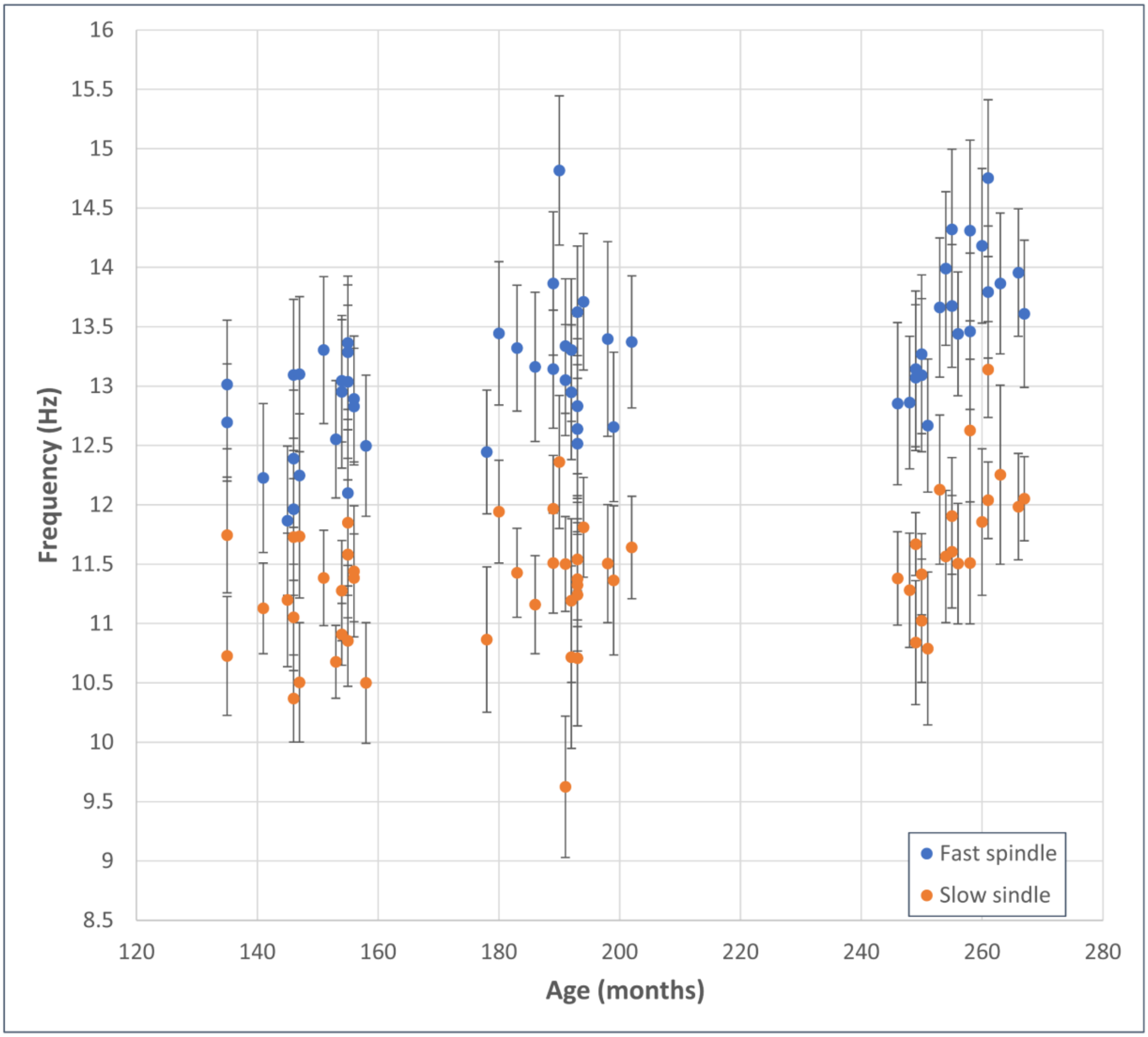

Supplementary Figure 2. Age-related individual fast and slow spindle frequency ranges (frequency thresholds) and the midpoints of the frequency ranges. The individual frequency ranges (black bars) were determined by the IAM method for the 60 subjects included in the analyses. Blue dots mark the midpoints of the fast spindle and orange dots mark the midpoints of the slow spindle frequency ranges.

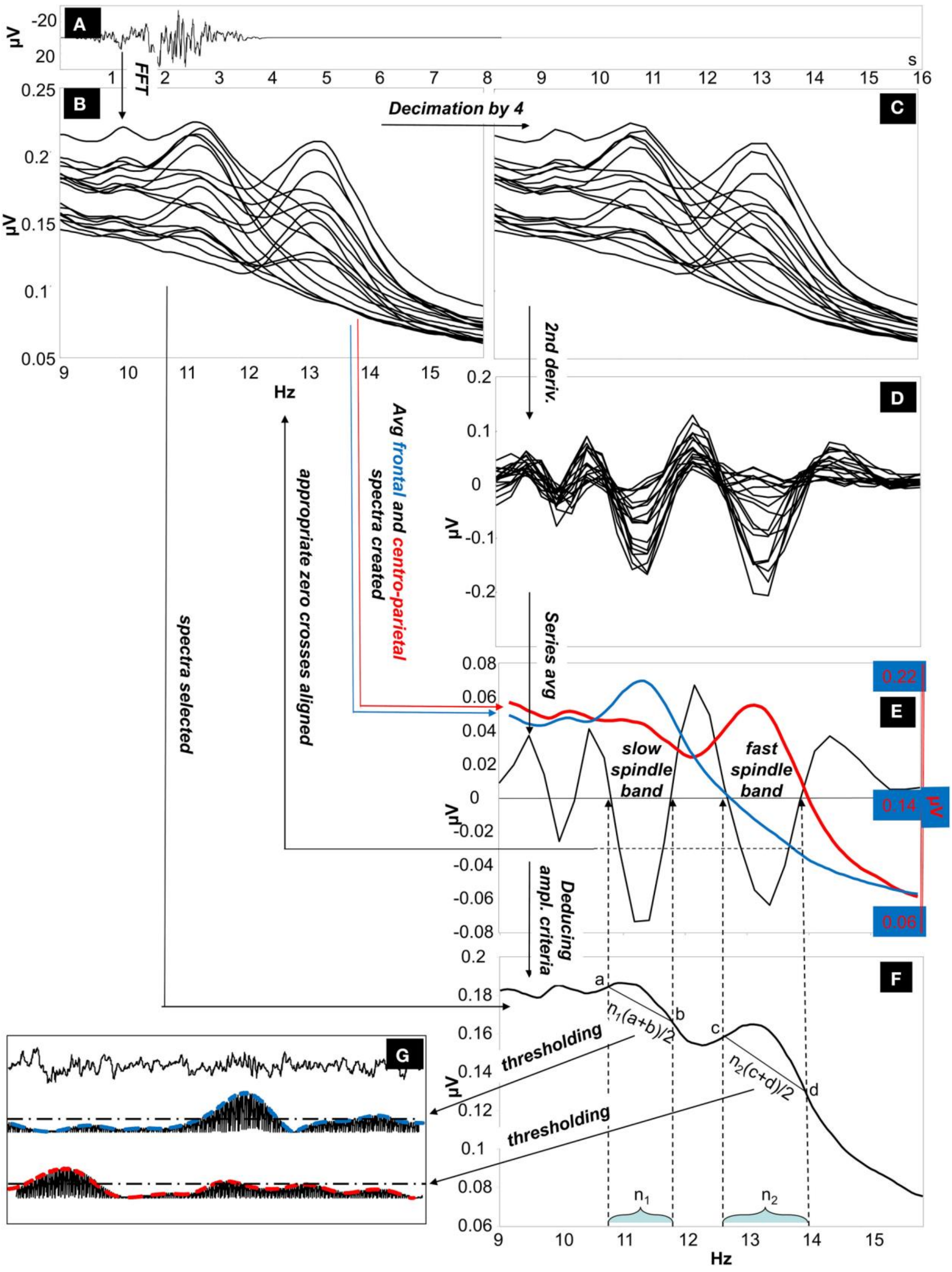

Supplementary Figure 3. The Individual Adjustment Method (IAM) of sleep spindle analysis. (A) Four-second EEG epoch Hanning-tapered and zero padded to 16 s. (B) Fast Fourier Transformation (FFT) is used to calculate 9–16 Hz average amplitude spectra of all night NREM sleep EEG from Hanning-tapered and zero-padded segments (derivations:

Fp1, Fp2, F3, F4, Fz, F7, F8, T3, T4, T5, T6, C3, C4, Cz, P3, P4, O1, O2 referred to the mathematically-linked mastoids). (C) Amplitude spectra are decimated (down-sampled) by a factor of 4. (D) Second order derivatives of the decimated amplitude spectra. (E) Calculating the whole-scalp second order derivatives by averaging all series. The resulting average series is overplotted with the averaged frontal (Fp1, Fp2, F3, F4, Fz, F7, F8) and centro-parietal (C3, C4, Cz, P3, P4) amplitude spectra (the left-side Y axis is for average second-order derivatives, while the second Y axis on the right is for average amplitude spectra). Appropriate zero-crossing points encompassing individual-specific slow and fast sleep spindle bands are selected on the 9–16 Hz frequency scale. (F) Derivation-specific amplitude criteria are calculated. (G) Thresholding of the envelopes of the slow and fast-spindle filtered signal.

Reproduced from Ujma et al., 2015. (https://doi.org/10.3389/fnhum.2015.00052). The work is available under Creative Commons Attribution license (CC BY 4.0) (http://creativecommons.org/licenses/by/4.0/). No changes were made.
